# Brain Imaging in Epilepsy-Focus on Diffusion-Weighted Imaging

**DOI:** 10.3390/diagnostics12112602

**Published:** 2022-10-27

**Authors:** Tzu-Hsin Huang, Ming-Chi Lai, Yu-Shiue Chen, Chin-Wei Huang

**Affiliations:** 1 Department of Neurology, National Cheng Kung University Hospital, College of Medicine, National Cheng Kung University, Tainan 70142, Taiwan; 2 Zhengxin Neurology & Rehabilitation Center, Tainan 70459, Taiwan; 3 Department of Pediatrics, Chi-Mei Medical Center, Tainan 71004, Taiwan

**Keywords:** diffusion-weighted imaging, epilepsy, seizure, ictal, magnetic resonance imaging, tractography

## Abstract

Epilepsy is a common neurological disorder; 1% of people worldwide have epilepsy. Differentiating epileptic seizures from other acute neurological disorders in a clinical setting can be challenging. Approximately one-third of patients have drug-resistant epilepsy that is not well controlled by current antiepileptic drug therapy. Surgical treatment is potentially curative if the epileptogenic focus is accurately localized. Diffusion-weighted imaging (DWI) is an advanced magnetic resonance imaging technique that is sensitive to the diffusion of water molecules and provides additional information on the microstructure of tissue. Qualitative and quantitative analysis of peri-ictal, postictal, and interictal diffusion images can aid the differential diagnosis of seizures and seizure foci localization. This review focused on the fundamentals of DWI and its associated techniques, such as apparent diffusion coefficient, diffusion tensor imaging, and tractography, as well as their impact on epilepsy in terms of differential diagnosis, epileptic foci determination, and prognosis prediction.

## 1. Introduction

Epilepsy is a chronic disease of the brain, which not only increases morbidity and mortality but also profoundly affects patients’ quality of life, education, and career. The prevalence of epilepsy [1] was estimated to be 6.38 per 1000 persons, affecting approximately 70 million people worldwide, with a lifetime prevalence of 7.60 per 1000 persons. Although 60–70% of patients with epilepsy can achieve satisfactory control through antiepileptic drug (AED) therapy alone, 13.6–25% have drug-resistant epilepsy (DRE), necessitating additional nonpharmacological therapies such as epilepsy surgery, ketogenic diet, vagus nerve stimulation, and cortical stimulation [2,3].

Brain imaging plays a vital role in the diagnosis and treatment of epilepsy, especially when epilepsy surgery is being considered for a patient with DRE. Advancements in magnetic resonance imaging (MRI) technology, acquisition protocols, and image processing methods have led to many improvements in brain imaging of epileptic areas in terms of the identification of seizure origin and etiology and preoperative evaluation. Diffusion-weighted imaging (DWI) is highly sensitive to early neuronal damage in stroke and seizures. DWI-associated technologies include apparent diffusion coefficient (ADC), diffusion tensor imaging (DTI), and fiber tractography, all of which play vital roles in the aforementioned tasks.

In this review, we focused on the development of DWI and its associated techniques, such as DTI, ADC, and tractography, as well as their impact on the diagnosis and treatment of epilepsy, including epilepsy surgery. We begin with the history of the development of DWI and the concept of this technique. We then review the current studies on transient DWI changes in epilepsy in clinical practice, which is followed by a summary of DWI and related techniques in epilepsy surgery. Finally, we discuss some images of cases of epilepsy with DWI changes in different conditions from our hospital, National Cheng Kung University Hospital, in Tainan, Taiwan.

## 2. Scope of Review

**Figure 1:**
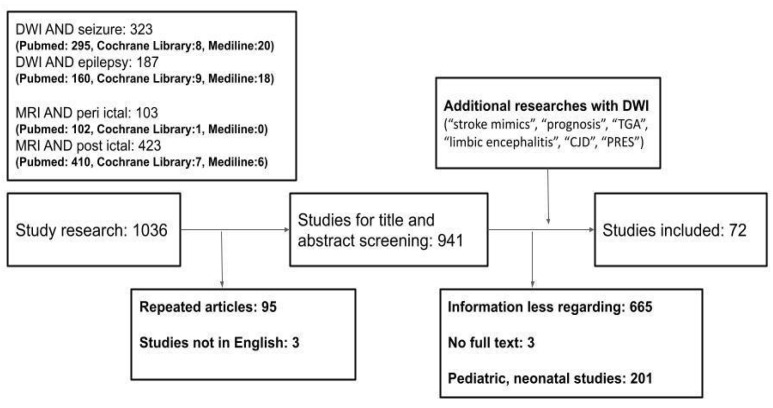
Database search flowchart. DWI, Diffusion-weighted Imaging; CJD, Creutzfeldt–Jakob disease; PRES, Posterior reversible encephalopathy syndrome; ADC, Apparent diffusion coefficient; TGA, transient global amnesia; MRI, magnetic resonance imaging.

We searched PubMed, MEDLINE, and LANCET databases for English-language, peer-reviewed journal articles, including original articles, case reports, clinical trials, reviews, meta-analyses, and systematic reviews, published between 1995 and 31 July 2022. We excluded letters because of the insufficient amount of information. The main search terms were “diffusion-weighted imaging OR Magnetic Resonance Imaging” AND “seizure OR epilepsy”. Additional key search terms included “peri-ictal”, “postictal”, “interictal”, “stroke mimics”, “epilepsy surgery”, “prognosis”, “epileptogenic foci”, “TGA”, “limbic encephalitis”, “CJD”, “PRES”. After duplicates were removed from the search results, the titles and abstracts of the remaining studies were screened for potential eligibility. Next, full texts of the articles considered for inclusion were screened, and their references were checked for additional studies, if necessary. Articles that did not address information between DWI and seizure and epilepsy were excluded. A total of 72 articles were included in the review (Figure 1).

## 3. DWI: Basic Concept

DWI is an advanced magnetic resonance technique that has revolutionized diagnostic imaging. The origin of DWI can be traced back to concepts proposed by Carr and Purcell, who observed nuclear magnetic resonance effects on molecular diffusion in 1954 [4]. In 1965 [5], Stejskal and Tanner established the basis of image acquisition methods used in modern clinical DWI MR by applying short-duration gradient pulses to obtain diffusion sensitization. However, the requirement for hardware and software delayed its introduction into routine clinical practice until the mid-1980s [6]. In 1986, Wesbey reported the early medical imaging of healthy and diseased brains by using DWI, thus establishing its utility in neuroradiology [7].

DWI measures the diffusion restriction of water molecules. In a perfectly homogenous environment without barriers, the diffusion of water has equal probability in all directions, which is known as Brownian motion and refers to the random motion of molecules [8]. In a complex environment of the human body, such as the brain, the normal free diffusion of water molecules is confined owing to the presence of cell membranes or myelin, macromolecules that interrupt the diffusion of smaller molecules, and microcirculatory effects [9,10]. Mapping the degree of water diffusion restriction enables us to establish the microstructure at the cellular level and therefore characterize cells in pathological processes with altering biophysical properties [11].

DWI presents a quantitative, two-dimensional map that displays the spatial distribution of the diffusion rate of water within the brain. Unlike the mechanisms in T1 and T2 relaxation, every pixel value in DWI presents diffusivity in these voxels (volume pixel). These voxels appear hypointense with high diffusion (e.g., cerebrospinal fluid) and hyperintense with low diffusion (e.g., acute ischemic stroke) [12].

The ADC is quantified and indicated as the rate of diffusion in living systems, independent of T1 and T2 relaxation. The term “apparent” relates to the fact that this motion of molecules is also influenced by other physiological processes, such as heartbeat, breathing, or CSF pulsation. A high ADC value and hyperintensity on ADC maps indicate areas with a high rate of diffusion (e.g., CSF), whereas areas with restricted diffusion display ADC hypointensity (e.g., gray and white matter). ADC maps have the advantage of avoiding T2 effects that may mimic or obscure lesions on DWI. The ADC value is an absolute quantitative measurement of water motion that can be compared between series [13].

The signal intensity in DWI (*SDWI*) is influenced by proton density (*Pd*), echo time (TE), transverse relaxation time (T2), b-value (b), and ADC in the equation [14]:

*S_DWI_* = *k* × (*Pd*) × (*e*^−*TE*/*T2*^*e*^−*b*×*ADC*^)

where *k* is a constant and “b-value” denotes the sensitivity of the MR pulse sequence to diffusion effects. According to the equation, hyperintensity on T2-weighted images (that is, long T2) results in hyperintensity on DWI, whereas increased ADC results in hypointensity in DWI. The interpretation of clinical studies requires an understanding of these relationships. For example, hyperintensity in DWI may arise from the decreased ADC of an acute infarct and the increased T2 of a tumor [14].

Notably, because the diffusion gradients are added to a T2-weighted sequence during DWI, DWI is susceptible to various artifacts, such as T2 shine-through, T2 blackout, and T2 washout effects [14]. The T2 shine-through effect indicates that a high signal in DWI is not due to restricted diffusion but rather to a high T2 signal that shines through in DWI. DWI images should be compared to ADC values to confirm true restricted diffusion. In cases of true restricted diffusion, the region of increased DWI signal demonstrates a low signal on ADC. On the other hand, T2 blackout is the opposite of the T2 shine-through phenomenon. In T2 blackout, lesions with very short T2 valued reduce the signal intensity in the DW images, potentially masking the diffusion sensitivity. The third phenomenon, called T2 washout, would appear with isointensity on DWI in combination of hyperintensity in T2WI and high ADC values. The T2 washout phenomenon could be seen in vasogenic edema in posterior reversible encephalopathy syndrome (PRES) or in the subacute phase of ischemic stroke.

Two main voxel parameters can also be extracted from DWI data: fractional anisotropy (meaning a preferred directionality of diffusion) and mean diffusivity (meaning the overall magnitude of diffusion). By combining the directional information and magnitude of anisotropic diffusion of each voxel, algorithms can visualize white matter tracts in the brain by following the pathways of unhindered water diffusion. This depends on the assumption that voxels with a similar direction of primary anisotropic diffusion are likely to be the same white matter tract. This is called tractography [10]. In tractography, major white matter tracts, such as corticospinal tracts, optic radiation, and arcuate fasciculus, are localized; tractography can thus help reduce damage during epilepsy surgery.

In summary, DWI has been widely used for detecting early ischemic infarction because of its higher sensitivity to early-onset pathophysiologic changes than that of T2-weighted images, even minutes after ischemic attacks [15]. It remains hyperintense within the first weeks after stroke [16,17]. In addition to ischemic lesions, DWI helps identify acute signal changes in other neurological diseases. Tractography provides useful information regarding the complex normal and abnormal neural networks and can guide the planning of brain tumor resection and postoperative neurological recovery.

## 4. DWI and Epilepsy

### 4.1. Cytotoxic, Ionic, and Vasogenic Edema

Cytotoxic edema is intracellular fluid accumulation without disrupting the blood–brain barrier [18]. It can occur through several mechanisms, such as the uptake of glutamate by a Na^+^-dependent mechanism and glutamate-induced AMPA receptor activation, resulting in Na^+^ entry [19]. The increased number of ions and neurotransmitters in cells creates osmotic gradients that lead to an influx of water through aquaporins and cell swelling. This causes hyperintensity in DWI and hypointensity in ADC (Table 1).

Vasogenic edema, which occurs because of blood–brain barrier disruption, leads to the diffusion of plasma from the vessel to interstitial space and causes fluid accumulation in the extracellular space [20]. Because of the increased diffusion rate of water in the interstitial space, the signals become hyperintense on the ADC. However, the signals become hyperintense in DWI as well because of the T2 shine-through effect from T2WI.

Notably, cytotoxic and vasogenic edema are not discrete events but a continuum of conditions. The third form of edema is ionic edema, which represents an intermediate transformation between cytotoxic and vasogenic edema.

**Figure 2:**
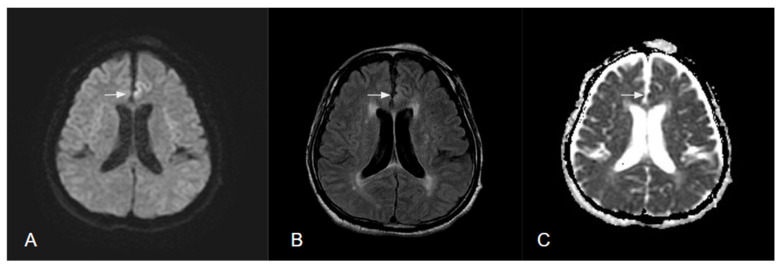
MR images of brain in a patient with status epilepticus. (**A**) Hyperintensity of DWI in cortical area and hypointensity in subcortical area. (**B**) Hyperintensity in T2 FLAIR at same region. (**C**) Hypointensity of ADC in cortical region.

Neuroimaging changes in brain edema in T2WI, DWI, and ADC indicate distinct spatiotemporal evolution and a mixture of cytotoxic, ionic, and vasogenic edema in the process of infarction, seizure, infection, or tumors (Figure 2).

### 4.2. Peri-ictal or Postictal MRI Changes

At the cellular level, interictal epileptiform discharge represents synchronous depolarization in a small neuronal pool. It then turns into highly repetitive discharges during seizure episodes and usually has sparse epileptiform discharge postictally. The ictal depolarizations may lead to a massive Na^+^ and Ca^2+^ influx, followed by water influx and K^+^ efflux, which may temporarily break down the Na^+^ /K^+^ adenosine triphosphatase pump. This causes a net translocation of water from the extracellular to intracellular space [20,21]. This is now the commonly accepted theory of reduced water diffusion which leads to high signals in DWI and a reduction in ADC.

Other findings have been observed in magnetic resonance spectroscopy (MRS). During seizures, the regional metabolic rate surges and results in higher blood flow to maintain the demand, but the oxygen supply remains insufficient, leading to lactate accumulation and worsening cytotoxic edema. Studies have observed increased DWI signals, decreased ADC, the appearance of lactate, and decreases in N-acetyl aspartate in MRS [22].

In 1997, Wieshmann et al. reported a case of a patient with status epilepticus and the associated changes in DWI and ADC [23]. DWI of this patient revealed common changes during focal status epilepticus—decreased diffusion in the cortex and increased diffusion in the associated subcortical white matter—and during transient paresis; these changes indicated resolution preceding functional recovery.

Most studies on single seizure or status epilepticus since have also revealed that the restriction of diffusion becomes normal after seizure cessation. This phenomenon is called seizure-induced reversible MRI abnormalities (SRMA).

### 4.3. SRMA

#### 4.3.1. Duration

The duration of normalization in DWI after seizure activities was explored in a prospective study in 2006 [24]. The researchers reported that the diffusion abnormality returned to the interictal levels in a median of 46 min after the initial postictal scan. Notably, their results also suggest that the diffusion abnormality in single seizures occurs more rapidly and more transiently than in status epilepticus (SE).

In 2018, a German retrospective study on MRI performed within 24 h after seizure occurrence observed DWI restrictions in 19% of patients with SE or a series of seizures, in 3% after single focal seizures, and in 2.5% after single generalized seizures [25]. Although the signal changes appeared during the SE in some patients, the median time to SRMA appearance and resolution was 24 h and 96.5 days, respectively [26,27]. In patients with a single seizure, SRMA was observed as early as six hours from the seizure onset and resolved completely in as early as five days [28,29].

#### 4.3.2. Frequency

The frequency of detected MRI abnormalities varied considerably, between 0.007% after a single seizure or seizure clustering [24,30] and 29.4% after SE [24,31,32], possibly because of the data heterogeneity, retrospective design, and limited sample size of these studies.

#### 4.3.3. Location

A 2021 systematic review [27] of cases with SE and MRI findings from 1987 to 2018 revealed five stereotypical features of SRMA involving the cortex, subcortical region, hippocampus, claustrum, and splenium. MRI abnormalities were mainly reported on T2-weighted sequences, followed by diffusion-weighted images. However, these locations can vary depending on etiologies and epileptogenic foci.

Studies have reported that the medial pulvinar is highly involved in temporal lobe SE, with approximately 54.5% of patients exhibiting DWI restriction, possibly through the cortico-thalamic network [33]. DWI abnormalities were observed more in the left temporal SE than in the right temporal SE.

When associated with electroencephalography (EEG), these peri-ictal DWI changes are often associated with ipsilateral EEG abnormalities in SE [34,35] or with ipsilateral lateralized periodic discharges (LPD) [36], although interpreting LPD as an interictal or peri-ictal pattern remains difficult [37].

#### 4.3.4. Localization

Focal cortical dysplasia, hippocampal sclerosis, polymicrogyria, and ganglioglioma are typical structural lesions in patients with drug-resistant focal epilepsy. Their detection and precise mapping before surgery are critical to determining whether these lesions are part of the epileptic zone; otherwise, their incomplete removal can result in seizure recurrence. This, however, is challenging in patients with focal epilepsy without visible lesions on MRI. Generally, in patients with newly diagnosed focal epilepsy, 18.7% have MR abnormalities that are considered epilepsy related [38].

One study indicated that DWI changes, if scanned within 150 min of EEG-documented seizures, could have localization value in temporal lobe epilepsy [37]. Nevertheless, as the change in DWI is less likely to be persistent, its clinical value is uncertain.

#### 4.3.5. SE

SE is easier to diagnose in emergency departments, but differentiating patients with first-time seizures and those in the postictal state from those with infarction may be difficult. Brain MR images may help in the differential diagnosis.

A retrospective review of 431 patients in 2017 provided a general view of MRI abnormalities in patients arriving at the ER with seizures [30]. They observed that 11 of 69 patients (15.9%) with first-time seizures in the emergency room had transient changes in the MRI. Of these, seven (63.6%) were diagnosed as having SE. The topography of these signal changes tends to involve the hippocampus, thalamus, and cortex, especially the ipsilateral side. This implies high network connectivity between these structures, making them all simultaneously vulnerable during the ictal phase. However, the cortex involved can extend beyond the vascular territory. Finally, these regions tend to exhibit hyperperfusion.

Another similar German study yielded the same conclusion. They reported DWI alterations in 3% of patients with single focal seizure, 19% in all patients with SE, and up to 56% in patients with focal SE. They implied that the restrictions observed in DWI after a single seizure tend not to present as a classical garland-like pattern in the cortex, and they were also much smaller and challenging to detect [25].

Excitotoxicity and edema can sometimes lead to permanent cellular damage and irreversible change [36,39]. As a result, more variable DWI findings are observed in patients with SE than in those with a single seizure [40], as a mixture of cytotoxic and vasogenic, reversible, and irreversible changes are present.

#### 4.3.6. Is DWI Necessary in Routine MRI in Epilepsy?

Structural MRI is fundamental for diagnosing and treating generalized and focal epilepsy. To broadly identify etiologies in DRE, the Neuroimaging Task Force recommends the use of the Harmonized Neuroimaging of Epilepsy Structural Sequences (HARNESS-MRI) protocol, as these sequences are available on most MRI machines regardless of the clinical setting and country. This protocol suggests using isotropic, millimetric 3D T1 and FLAIR images and high-resolution 2D sub-millimetric T2 images to obtain enough information.

DWI is not necessary for all patients with epilepsy undergoing epilepsy presurgical evaluation, but DWI-derived parameters can provide further information for certain pathologies such as hippocampal sclerosis. The purposes of DWI used in epilepsy nowadays mainly focus on two things: (A) distinguishing epilepsy from other etiologies or (B) white matter network evaluation before and after surgery.

## 5. DWI and Epilepsy Surgery

The goal of epilepsy surgery is to obtain permanent seizure freedom while causing no or minimal deficit of functions. This requires a detailed and comprehensive survey of the associated cortex, white matter tracts, and networks involved in the surgery. During the preoperative evaluation, the delineation of language, motor, and visual cortexes and their white matter tracts is essential if these structural lesions are close. Now, accumulative data indicate the high reliability of functional MRI and DTI techniques in achieving this goal.

Unlike fMRI, used for identifying the eloquent cortex, DTI is a technique to measure the diffusivity of water molecules and thus enables visualization and evaluation of the integrity of the tract. The value of DTI depends on the mean diffusivity (MD) and fractional anisotropy (FA). MD provides the overall magnitude of water diffusivity, and FA represents the dominant direction of water motion. DTI tractography connects a nearby voxel with a similar direction of water diffusion and then generates the tracts within the white matter. At least 90% of voxels in the brain contain more than one fiber direction [41,42]; therefore, researchers are studying more complex analytical models that do not fit the simplified assumption that only one major fiber direction exists in one voxel.

### 5.1. Motor Function Evaluation

Tractography, along with fMRI, is implemented in the preoperative evaluation of the corticospinal tract (CST) [43]. Previously, CST was believed to originate mainly from the precentral gyrus, the primary motor cortex, whereas, in 2009, a study [44] indicated that the origin of CST is located in both pre- and post-central gyri in 71% of healthy children rather than confined to the precentral gyrus. In addition, the CST is modified either in origin or track if early structural abnormalities occur. The identification of CST with tractography can be beneficial in frontal lobe surgery, especially for children for whom undergoing fMRI would be challenging. One study [45] revealed similar localization and risk-predictive values in tractography and invasive electrical stimulation mapping.

### 5.2. Visual Field Evaluation

Temporal lobe epilepsy is a seizure originating in the temporal lobe, and anterior temporal lobe resection combined with amygdalohippocampectomy remains one of the most effective treatments. Nevertheless, more than half of patients undergoing these operations develop visual field deficits [46].

Many clinical studies have focused on optic radiation damage, especially Meyer’s loop during anterior temporal lobe resection, which can cause profound visual field deficit [47]. The display of tractography during surgery and the use of intraoperative MRI can reduce the risk of visual field deficit.

### 5.3. Language Evaluation

The major structure of interest in the language network is the arcuate fasciculus, which connects Wernicke’s and Broca’s areas. It is easily damaged during frontotemporal resection surgery. However, the arcuate fasciculus is not the only structure in the network. The condition becomes more complicated because the complex language pathway involves the premotor cortex and inferior parietal regions [48,49,50]. These pathways can be more atypical and harder to recognize because of early brain injury, malformation, or chronic epilepsy, leading to reorganization [51].

In addition to the current gold-standard method of using electrical stimulation mapping, some alternatively noninvasive and approachable methods, such as the DWI-MAP classifier, have been established, which exhibits 77% accuracy in normal children and 82% accuracy in children with focal epilepsy for predicting language activation areas [52].

### 5.4. Presurgical Differential Diagnosis

Differential diagnosis before surgery is critical to guiding treatment strategy. In children, focal cortical dysplasia, dysembryoplastic neuroepithelial tumors, and gangliogliomas share similar clinical features and cannot be easily distinguished using conventional MRI alone. Combining conventional MRI, ADC, and MRS facilitates separation [53].

## 6. Clinical Aspects of DWI in Epilepsy

### 6.1. Differential Diagnosis

#### 6.1.1. Acute Ischemic Stroke

In the emergency room, epileptic seizures are a leading cause of stroke mimics. Since the 19th century, seizures have been recognized as a cause of focal paresis, and Todd’s paresis can be short, mimicking transient ischemic attack [54]. Postictal aphasia has been documented after a dominant hemisphere seizure [55]. These deficits can last for days in the case of generalized seizures. Hence, if seizure episodes (especially the ictal phase) are not witnessed, these conditions can be mistakenly identified as vascular events.

A 2021 systematic review [54] included 61 studies with 62,664 patients with stroke and stated that the stroke mimic rate was nearly 25%, with 13% of stroke mimics involving epileptic seizures. Although some similarities exist in the initial clinical symptoms of stroke and epileptic seizures, the underlying mechanisms and physiological changes differ, as do the MRI changes.

**Figure 3:**
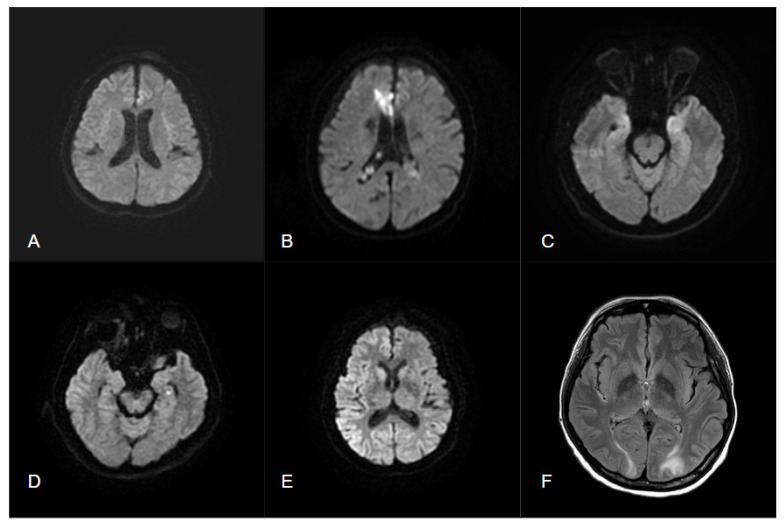
(**A**) Status epilepticus presents a left frontal cortical hyperintensity in DWI; (**B**) acute ischemic infarction in right ACA territory with DWI hyperintensity; (**C**) autoimmune encephalitis presents with bilateral hyperintensity in DWI and T2 FLAIR; (**D**) dot-like hyperintensity of DWI in TGA; (**E**) diffuse gyriform hyperintensity of DWI in cerebral cortex in sCJD; (**F**) bilateral hyperintensity of T2 FLAIR in white matter regions without abnormalities in DWI in PRES.

DWI hyperintensity and ADC hypointensity can be observed within one hour of middle cerebral artery occlusion, followed by T2WI hyperintensity appearing 2–3 h later, which indicates irreversible changes due to cell death. Differentiating an epileptic seizure from early cortical ischemic stroke through MRI alone is challenging. However, some changes, such as nonvascular distribution, absent vascular occlusion, and normal or increased perfusion, suggest epileptic origin (Table 2 and Figure 3).

DWI reversal can also be observed after early treatment with tPA or endovascular therapy because of reperfusion of the ischemic tissue [56,57]. This may be more common in patients with epilepsy; however, DWI reversal in ischemia tends to occur in the penumbra area, whereas in epilepsy, it tends to occur in the cortex and associated network structures.

Notably, seizures accompany ischemic stroke, especially when cortical regions are involved in embolic stroke. Approximately 3.8% of all patients with stroke had an early seizure (within 7–14 days of stroke), whereas only 1.5% had a seizure upon stroke onset [58]. In this case, DWI changes are more likely due to ischemia than to seizure itself.

#### 6.1.2. Transient Epileptic Amnesia and Transient Global Amnesia

Transient global amnesia is a distinct clinical syndrome with sudden onset and dramatic anterograde amnesia lasting mostly less than 24 h. The underlying mechanism remains unknown, but it was believed to be a transient disturbance to specific hippocampal circuits in memory processing [59,60]. However, transient epileptic amnesia (TEA) syndrome can be a much more underdiagnosed disease, especially when it presents only slight or no typical epileptic manifestations. TEA is believed to be due to focal ictal discharges, usually within the temporal lobe.

These two disorders present with similar symptoms initially and differentiating them is challenging at first. A 2018 retrospective study in Italy revealed that patients with TEA tend to have a recurrence of symptoms, confusion state, or language disturbance. No significant difference in the symptom duration between TGA and TEA was observed, even though symptoms are believed to last shorter in TEA. The 24 h EEG revealed great values in TEA, especially during sleep. DWI in MR images indicated typical lateral hippocampus dot-like hyperintensity in TGA and less in patients with TEA. A 2021 retrospective study including 201 patients with TGA concluded that the detection rates were highest 2–4 days after symptom onset by using DWI and 2D/3D T2 FLAIR [61].

#### 6.1.3. Limbic Encephalitis

Limbic encephalitis means inflammation or infection that affects the limbic system, which must be differentiated from vascular or neoplasm etiologies. MRI remains the main neuroimaging technique for performing a more comprehensive brain analysis. In a retrospective review of 251 suspected cases of encephalitis with temporal lobe abnormalities in MRI [62], herpes simplex encephalitis comprised nearly 25% of cases. Unilateral rather than bilateral temporal lobe changes, insular involvement, and the absence of basal ganglia involvement suggest herpes simplex encephalitis rather than autoimmune limbic encephalitis. The abnormalities are mostly observed in the T2WI sequence.

The use of DWI images also helps to further distinguish seizure occurrence from limbic encephalitis [63]. In 2020, a study conducted by Mayo Clinic [64] identified two patterns of DWI abnormalities specific to seizure activities (Table 2 and Figure 3): gyriform hippocampal diffusion restriction (Pattern 1) and diffuse hippocampal diffusion restriction that spares the most medial temporal lobe structures (Pattern 2). These findings can raise concern regarding seizures, even in patients with limbic encephalitis, and highlight the need for AEDs to prevent further neuronal damage because of repetitive seizures.

**Table 2:** Summary of common neurological disorders associated with DWI changes.

	DWI Location	DWI Change in Time	T2 FLAIR	Other Features
Epilepsy	- Commonly involves (1) cortex, (2) subcortical region, (3) hippocampus, (4) claustrum, (5) splenium (6) pulvinar - Nonvascular distribution	Hyperintensity can occur within hours and resolve within days to weeks depending on severity and etiologies	Hyperintensities may appear if structural lesions exist or irreversible cell death occurs	Absent vascular occlusion
Ischemic stroke	Distributed within vascular territories	Hyperintensities can occur within minutes and reach a peak after days before gradually reducing	Hyperintensities persist in the ischemia sites for years	DWI reversal may occur after early reperfusion with t-PA or endovascular therapy
limbic encephalitis	May involve uncus, amygdala, hippocampus	Nonspecific patterns (requires further research)	Autoimmune: bilateral mesial temporal lobe Infectious: unilateral temporal lobe, insular involvement, absence of basal ganglion.	Two patterns raise concerns regarding seizure: (A) gyriform hippocampal restriction and (B) diffuse hippocampal diffusion restriction that spares the most medial temporal lobe structures
Transient global amnesia	- Dot-like hyperintense lesions 1–2 mm in diameter - Commonly in the lateral aspect of the hippocampus (CA-1 region)	Hyperintensity appears with a delay of 24–48 h after onset	Hyperintensities highly detected 2–4 days after onset	
CJD	- Mainly affects striatum and cortex - Gyriform hyperintensity correlates to location of periodic sharp-wave complex on EEG	Hyperintensity appears 0.5–7 (mean: 1.6) months after symptoms onset, is progressive and persistent over months, then disappears in the end phase	Isointensity to mild hyperintensity (DWI > T2 FLAIR)	Pulvinar sign (symmetrical hyperintensity in the posterior thalamic nuclei on DWI or T2WI) or hockey stick sign (affecting the dorsomedial thalamic nuclei) specific in vCJD, sometimes in sCJD
PRES	Similar to T2WI	Hypointense to isointense	- Hyperintensities appear in the parieto-occipital, posterior frontal cortex, white matter - Less commonly in the brainstem, basal ganglia, and cerebellum	DWI and ADC hyperintensity indicate vasogenic edema

DWI, Diffusion-Weighted Imaging; T2 FLAIR, T2-weighted-Fluid-Attenuated Inversion Recovery; CJD, Creutzfeldt–Jakob disease; EEG, Electroencephalography; vCJD, variant Creutzfeldt–Jakob Disease; sCJD, sporadic Creutzfeldt–Jakob Disease; PRES, Posterior reversible encephalopathy syndrome; ADC, Apparent diffusion coefficient.

#### 6.1.4. Creutzfeldt–Jakob Disease

Prion diseases, previously known as transmissible spongiform encephalopathy, are fatal neurodegenerative diseases that affect both human and nonhuman mammals. Creutzfeldt–Jakob disease (CJD) is the most common human prion disease worldwide and can also be classified as sporadic, acquired, or familial [65]. Typical sCJD cases involve a rapidly progressive clinical course with psychiatric, visual, and memory disturbance in the early phase, cognition dysfunction and myoclonus in the later phase, and akinetic mutism several months after disease onset.

MRI is useful for the diagnosis of CJD. In the early phase, DWI hyperintensity appears in the cerebral cortex and striatum. Often, the gyriform hyperintensity correlates to the location of the periodic sharp-wave complex on EEG [66]. The average duration between disease onset and the first observation of cerebral cortical hyperintensity on DWI was 1.6 ± 1.3 months. This DWI hyperintensity of gray matter indicates the pathology of the spongiform change in CJD. These hyperintensity regions gradually diminish and finally disappear in the end [67]. Compared with epilepsy, the DWI changes in CJD tend to last longer, have broader involvement of the cortex, and disappear gradually in the late phase (Figure 3).

#### 6.1.5. Posterior Reversible Encephalopathy Syndrome

Posterior reversible encephalopathy syndrome (PRES) is another critical disorder that must be identified in the early stage [68]. It is a (sub)acute condition characterized by various neurological symptoms, including headache, impaired visual acuity or visual field deficits, impaired consciousness, confusion, seizures, and focal neurological deficits. Most patients present with elevated arterial blood pressure, in some cases hypertensive emergencies.

The typical imaging findings of PRES include hyperintensity on T2 FLAIR in the areas of the parieto-occipital, posterior frontal cortex, and white matter and, less commonly, in the brainstem, basal ganglia, and cerebellum (Figure 3). It may be a form of intense vasogenic edema, and it appears as hypo- to isointense signals on DWI and hyperintense signals on ADC mapping [69]. The overall prognosis is favorable because clinical symptoms and imaging lesions are reversible in most patients [70].

### 6.2. As a Prognostic Marker

Studies have evaluated whether MRI changes, such as those in DWI, can predict seizure prognosis. In a retrospective single-center study involving patients with SE, the restriction of DWI and signal abnormalities in T2WI were associated with poorer functional conditions at discharge [71]. In patients with herpes simplex virus encephalitis, diffusion restriction was also considered a marker of unfavorable outcomes [72].

## 7. Conclusions

DWI is an advanced technique in MRI. Several sequences based on DWI, such as tractography and DTI, provide further information that helps determine epileptogenic foci and networks for epilepsy surgery. Although the precise frequency and duration of reversible DWI changes in patients with single seizure or status epilepticus are unclear at this stage, studies have discovered that the etiologies, severity, and duration of seizure are associated with distinct, more detectable, and sustained DWI changes. With the aid of DWI changes in different disease entities, such as ischemic infarction, epilepsy, encephalitis, TGA, CJD, and PRES, we can differentially diagnose these major neurological disorders and understand the role of DWI in epilepsy. Studies combining sequences in MRI with potential artificial deep learning algorithms may aid significantly in the identification of epileptic focus in patients with epilepsy.

## Figures and Tables

**Table 1:** Three types of MRI abnormalities in DWI, ADC, and T2WI sequences.

	DWI	ADC	T2WI	Mechanism
Cytotoxic edema (acute phase ischemic infarction), abscess, demyelination	↑	↓	?	Diffusion restriction
CSF, chronic phase ischemic infarction	↓	↑↑	↑	Increased water diffusion
Vasogenic edema	↑	↑	↑	T2 shine through

CSF, cerebrospinal fluid; DWI, diffusion-weighted Imaging; T2 FLAIR, T2-weighted fluid attenuated inversion recovery; ADC, apparent diffusion coefficient. ↑ indicates hyperintensity, down arrow; ↓ indicates hypointensity; ? indicates variable level of cellular death in diseases process.

## Data Availability

Data are available upon request from the correspondence authors.
